# Association of the Primary Care Frailty Index with Postoperative Outcomes in Older Patients Undergoing Major Gastrointestinal Oncologic Surgery: A Retrospective Cohort Study

**DOI:** 10.3390/jcm15135158

**Published:** 2026-07-02

**Authors:** Andrea Costanzi, Nicola Fazzini, Lara Verdi, Paolo Dionigi Rossi, Marco Enoc Chiarelli, Giulia Bonfanti, Paolo Aseni

**Affiliations:** 1 General Surgery Unit, San Leopoldo Mandic Hospital, ASST Lecco, 23807 Merate, Italy; n.fazzini@asst-lecco.it (N.F.); m.chiarelli@asst-lecco.it (M.E.C.); giu.bonfanti@asst-lecco.it (G.B.); 2 Internal Medicine Unit, San Leopoldo Mandic Hospital, ASST Lecco, 23807 Merate, Italy; l.verdi@asst-lecco.it (L.V.); pa.rossi@asst-lecco.it (P.D.R.); 3 Department of Emergency Medicine GOM Niguarda, Niguarda Hospital, 20162 Milan, Italy; paolo.aseni@ospedaleniguarda.it

**Keywords:** frailty assessment, primary care frailty index, gastrointestinal cancer surgery, perioperative risk stratification, postoperative outcomes

## Abstract

**Background/Objectives:** Frailty is increasingly recognized as a major determinant of postoperative outcomes in older patients undergoing oncologic surgery. The Primary Care Frailty Index (PC-FI), a deficit accumulation-based instrument derived from routinely available clinical information, has recently been proposed as a practical frailty assessment tool. However, evidence supporting its application in gastrointestinal surgical oncology remains limited. **Methods:** We conducted a retrospective cohort study including patients aged ≥65 years who underwent elective major colorectal or gastric cancer surgery between January 2022 and October 2025 at a tertiary Italian hospital. Patients with a PC-FI > 0.07 were included and categorized as having mild (0.07–0.13) or moderate-to-severe frailty (≥0.14). Postoperative outcomes included the Comprehensive Complication Index (CCI), Clavien–Dindo classification, and length of hospital stay. Receiver operating characteristic (ROC) analysis was performed to compare the discriminative performance of PC-FI, ASA classification, and the ACS Surgical Risk Calculator. **Results:** Ninety-two patients met the inclusion criteria. Patients with moderate-to-severe frailty were significantly older and had higher ASA class, Charlson Comorbidity Index, and ACS morbidity estimates than mildly frail patients. They also experienced a greater postoperative complication burden (mean CCI 25.96 vs. 16.40, *p* = 0.02) and longer hospital stay (9.89 vs. 7.43 days, *p* = 0.01). ROC analysis demonstrated modest discriminative performance for PC-FI (AUC 0.63), comparable to ASA classification (AUC 0.68) and the ACS morbidity score (AUC 0.70), without statistically significant differences among the three instruments. **Conclusions:** Higher PC-FI scores were associated with increased postoperative morbidity and prolonged recovery following major gastrointestinal oncologic surgery. Although its discriminative performance was modest and does not support its use as a stand-alone risk prediction tool, the PC-FI may represent a simple first-line frailty screening instrument to identify older patients who could benefit from comprehensive multidisciplinary perioperative assessment.

## 1. Introduction

Frailty has emerged as one of the most important determinants of postoperative outcomes in older patients undergoing major surgery. Rather than representing the simple consequence of chronological aging, frailty reflects a multidimensional state of reduced physiological reserve and increased vulnerability to surgical stress, resulting from the cumulative decline of multiple biological systems. Numerous studies [1,2,3,4,5] have demonstrated that frailty is associated with a higher incidence of postoperative complications, prolonged hospitalization, delayed functional recovery, institutionalization, and mortality across a wide range of surgical specialties, particularly in gastrointestinal oncologic surgery. From a pathophysiological perspective, frailty is characterized by multisystem dysregulation involving the musculoskeletal, immune, metabolic, and neuroendocrine systems [6,7]. Sarcopenia plays a pivotal role as a biological substrate, contributing to reduced strength, impaired mobility, and diminished resilience to physiological stress [8,9,10]. These physical alterations are frequently accompanied by cognitive impairment, depression, and social vulnerability, further reinforcing the multidimensional nature of frailty [11,12]. Notably, frailty is a dynamic condition: individuals may transition between states of robustness, pre-frailty, and frailty over time, which opens opportunities for targeted interventions, including prehabilitation strategies in the perioperative setting [13,14,15]. The global demographic transition toward an aging population has made frailty a major public health concern. According to United Nations estimates, the number of individuals aged 65 years and older is expected to more than double worldwide, rising from approximately 770 million in 2020 to over 1.5 billion by 2050 [16]. This trend is particularly pronounced in high-income countries but is also rapidly accelerating in low- and middle-income regions [17]. As life expectancy increases, the prevalence of age-related conditions, including frailty, is expected to rise substantially, placing significant pressure on healthcare systems [18]. Epidemiological studies indicate that frailty affects a considerable proportion of the older population, although prevalence estimates vary depending on the definition and assessment tools used [19]. In community-dwelling older adults, frailty prevalence typically ranges from 10% to 25%, while an additional 30–50% may be classified as pre-frail. In hospitalized or surgical populations, these figures are substantially higher due to patient selection and disease burden [19]. Importantly, frailty has consistently been associated with an increased risk of adverse outcomes, including postoperative complications, prolonged hospital stay, institutionalization, and mortality [5]. In the context of surgery, particularly oncologic surgery, frailty has gained increasing attention as a key determinant of perioperative risk [20]. Traditional risk stratification models based primarily on chronological age and comorbidities are often insufficient to capture the heterogeneity of older patients [21]. As a result, there is a growing emphasis on incorporating frailty assessment into preoperative evaluation to improve clinical decision-making and optimize patient outcomes [22,23]. To quantify this risk effectively, several instruments have been developed. The two most widely accepted conceptual models are the physical phenotype proposed by Fried et al., based on five clinical criteria, and the deficit accumulation model developed by Rockwood and Mitnitski, which conceptualizes frailty as the cumulative burden of multiple health deficits [3,4,5,6]. Although both models have been extensively validated, their implementation in routine surgical practice remains inconsistent because many assessment tools require dedicated geriatric evaluation, physical performance testing, or specialized personnel, limiting their feasibility in busy preoperative clinics [6,7]. Furthermore, despite the availability of multiple frailty instruments, their implementation in routine surgical practice remains inconsistent, partly due to complexity, time constraints, and lack of standardization [24,25]. In recent years, increasing attention has been directed toward the development of pragmatic frailty instruments that can be easily incorporated into routine clinical practice. Several frailty assessment tools, including the Clinical Frailty Scale (CFS), Modified Frailty Index (mFI), Edmonton Frail Scale, G8 screening tool, and Fried frailty phenotype, have demonstrated prognostic value in surgical patients. However, many of these instruments require dedicated assessments, specialized training, or additional patient-reported information, limiting their routine implementation in busy surgical settings. In contrast, the Primary Care Frailty Index is based on routinely available clinical data and may therefore offer a more pragmatic approach to perioperative frailty screening. Among these, the Primary Care Frailty Index (PC-FI), developed according to the deficit accumulation model, represents a particularly attractive approach because it relies primarily on routinely available clinical information collected during standard medical assessment [26]. Unlike more complex multidimensional geriatric evaluations, the PC-FI can be calculated using demographic characteristics, comorbidities, functional status, cognitive impairment, medication use, and other readily accessible health deficits, making it potentially suitable for rapid preoperative frailty screening.

## 2. Materials and Methods

This retrospective observational cohort study was designed to evaluate the association between preoperative frailty and short-term postoperative outcomes in older patients undergoing major oncologic surgery. The primary objective was to determine whether frailty, quantified using the PC-FI, could serve as a significant predictor of postoperative complications, length of hospital stay, and overall clinical course complexity.

### 2.1. Study Population

Patients aged ≥65 years who underwent elective major surgery for gastrointestinal malignancies (colon or gastric cancer) between January 2022 and October 2025 at the General Surgery Department of San Leopoldo Mandic Hospital, Merate (Italy) were retrospectively reviewed. Our institution utilizes a selective clinical protocol wherein only older patients displaying at least mild frailty characteristics (defined as a PC-FI score > 0.07) are automatically channelled into dedicated multidisciplinary perioperative care pathways. Consequently, patients aged ≥65 years presenting with a robust or non-frail baseline profile (PC-FI ≤ 0.07) were excluded from this analysis. Major surgical procedures were defined as operations associated with significant physiological stress, including both laparoscopic and open approaches (e.g., right/left hemicolectomies, segmental colonic resections, subtotal and total gastrectomies). Minor surgical procedures (such as cholecystectomy or simple abdominal wall repairs) were excluded. Based on these criteria, a final cohort of 92 consecutive patients was analyzed.

### 2.2. Frailty Assessment and PC-FI Calculation

The PC-FI was operationalized as a continuous deficit accumulation metric based on 30 distinct clinical items extracted from electronic health records. These items included 18 specific comorbidity variables (cardiovascular, respiratory, metabolic, and cerebrovascular conditions), 5 functional dependency markers (activities of daily living restrictions), 3 cognitive/neuropsychiatric components (documented dementia, depression, or history of confusion), polypharmacy thresholds (regular intake of ≥5 medications), and core baseline social support parameters. Each deficit was scored as 0 (absent) or 1 (present). For individuals with missing data, variables with greater than 5% missing entries across the cohort were completely omitted, and the individual index was calculated strictly as the ratio of actual deficits present to the total number of successfully recorded valid deficits. For clinical subgroup analyses, patients were stratified into two explicit severity tiers based on established criteria: mild frailty (PC-FI 0.07–0.13) and moderate-to-severe frailty (PC-FI ≥ 0.14).

### 2.3. Clinical Variables and Postoperative Outcomes

Preoperative data points included age, body mass index (BMI), Charlson Comorbidity Index, ASA classification, and the preoperative ACS Surgical Risk Score. Primary endpoints consisted of the length of postoperative hospital stay (LOS) and short-term postoperative complications. Postoperative complications were comprehensively tracked within a strict 90-day postoperative window and graded according to the Clavien–Dindo classification. Length of stay (LOS) was defined as the total number of inpatient postoperative hospitalization days from the date of the operative procedure until formal clinical discharge.

To accurately quantify the absolute cumulative morbidity burden of patients experiencing multiple concurrent adverse events, the Comprehensive Complication Index (CCI) was calculated for each patient on a continuous scale from 0 to 100. All data were extracted from electronic medical records, including preoperative assessments, operative reports, and postoperative clinical documentation. Data collection was conducted with careful attention to internal consistency and completeness, minimizing potential bias related to the retrospective design.

### 2.4. Statistical Analysis

Statistical analysis was performed using the open-source R statistical software (version 4.3.2; R Foundation for Statistical Computing Vienna, Austria; https://www.r-project.org/). Continuous variables were analyzed using Student’s *t*-test or the Wilcoxon–Mann–Whitney test depending on normality distributions. Categorical variables were evaluated via the Chi-square or Fisher’s exact test. To determine whether PC-FI functions as an independent marker of major postoperative morbidity, a multivariable logistic regression analysis was executed using a clinically relevant complication threshold defined as a CCI > 30. The model was adjusted for essential potential baseline confounders, including chronological age, ASA score, and the baseline ACS Risk Score estimate. Overall multivariable model calibration and discrimination were assessed using the Hosmer–Lemeshow goodness-of-fit test and Nagelkerke’s pseudo-R^2^ metric. The discriminative accuracy of PC-FI, ASA class, and ACS morbidity score was quantified via Receiver Operating Characteristic (ROC) curve analysis. All major area under the curve (AUC) estimates and clinical performance metrics (sensitivity, specificity, positive predictive value [PPV], and negative predictive value [NPV]) are reported with their respective exact 95% Confidence Intervals (95% CI). Comparisons among distinct ROC curves were executed using DeLong’s test. Regarding sample size considerations, the cohort size (*n* = 92) represents a complete census of the eligible clinical pathway population during the study timeframe. A post hoc power calculation demonstrated that this sample size provides approximately 80% power (α = 0.05) to detect a major difference in mean CCI scores between the frailty groups but is limited in detecting subtle statistical differences between closely related AUC curves. Statistical significance was set at *p* < 0.05.

## 3. Results

A total of 92 older patients were analyzed. The baseline demographic and clinical features of the entire cohort are presented in Table 1. The cohort exhibited a mean age of 79.07 years, with 80 patients (87.0%) undergoing surgery for colorectal malignancies and 12 patients (13.0%) for gastric cancer. The surgical approach was predominantly minimally invasive, with 68 procedures (73.9%) initiated laparoscopically. Based on the PC-FI screening thresholds, 42 patients (45.7%) presented with mild frailty, while 50 patients (54.3%) were classified as having moderate-to-severe frailty.

Comparative assessments between the two baseline frailty groups are detailed in Table 2. Patients in the moderate-to-severe frailty cohort were significantly older (median 81.5 vs. 76.0 years; *p* < 0.001), exhibited a substantially elevated systemic risk configuration (ASA III–IV: 84% vs. 31%; *p* < 0.001), and had higher cumulative baseline comorbidity indexes (median Charlson score 7.0 vs. 6.0; *p* < 0.001).

### 3.1. Postoperative Morbidity and Resource Utilization

**Figure 1:**
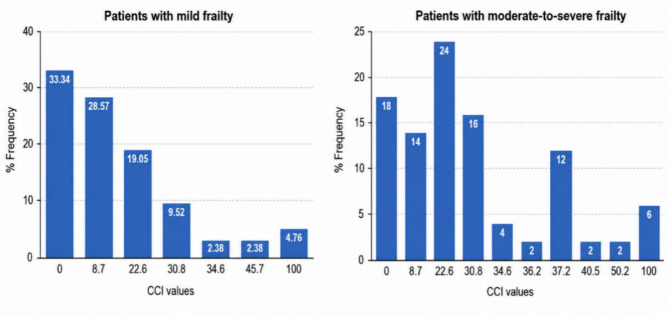
Distribution of CCI values in group of patients with mild and moderate-to-severe frailty.

A total of 23 patients (25.0%) achieved an entirely uncomplicated postoperative course (CCI = 0). However, patients classified with moderate-to-severe preoperative frailty experienced a significantly higher global complication burden than mildly frail peers, as evidenced by a significantly elevated mean CCI score (25.96 vs. 16.40; *p* = 0.02). Conversely, when postoperative complications were assessed purely as categorical events via standard Clavien–Dindo maximum grading thresholds, the difference failed to achieve statistical significance, underscoring the superior sensitivity of the continuous CCI metric in this older population (the distribution of CCI values is illustrated in Figure 1). Furthermore, the mean length of postoperative hospital stay was significantly prolonged in the moderate-to-severe frailty cohort (9.89 vs. 7.43 days; *p* = 0.01), suggesting that higher PC-FI values were associated with a delayed clinical recovery. The ACS morbidity risk score also differed significantly between the two frailty groups, with a mean score of 11.53% in the mild frailty group versus 19.95% in the moderate-to-severe cohort (*p* = 0.00053), confirming a strong alignment between the PC-FI index and established surgical risk models. A parallel analysis conducted by grouping patients into ASA I–II and ASA III–IV demonstrated consistent trends. The overall 90-day mortality rate was 5.4%, corresponding to 5 deaths, all occurring within the high-risk ASA III–IV subsets. Looking at the clinical details, two patients had right-sided colon cancer and underwent right hemicolectomy, one patient had substenosing transverse colon cancer and underwent transverse colon resection, and two patients had gastric cancer treated with subtotal gastrectomy. In two cases, death followed a surgical complication, namely anastomotic leakage. In the remaining three cases, clinical deterioration was related to medical infectious complications, including respiratory infections in two patients and endocarditis in one. According to the PC-FI classification, three deceased patients belonged to the moderate-to-severe frailty group, while two were in the mild frailty group.

### 3.2. Multivariable Logistic Regression Model

To isolate the independent association of preoperative frailty with major morbidity, a multivariable logistic regression model was constructed using major complications (CCI > 30) as the dependent variable (reported below in Table 3). After controlling for chronological age, ASA status, and the standard ACS Morbidity risk estimate, the continuous preoperative PC-FI score remained significantly associated with severe postoperative complications (adjusted Odds Ratio [aOR]: 1.32 per 0.05 index increment, 95% CI: 1.04–1.71; *p* = 0.024). The multivariable model demonstrated acceptable overall calibration (Hosmer–Lemeshow test *p* = 0.68) and reasonable overall fit properties (Nagelkerke R^2^ = 0.28).

### 3.3. Discriminative Performance and ROC Analysis

**Figure 2:**
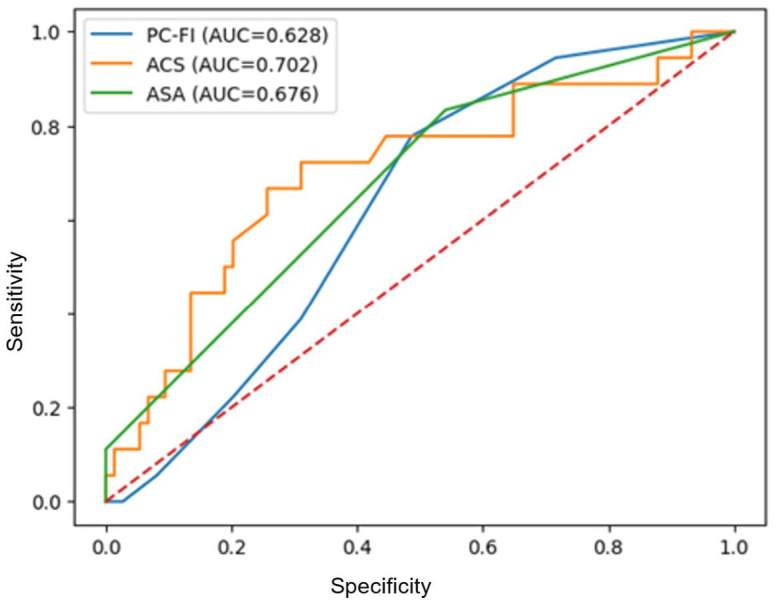
ROC curves for PC-FI, ASA and ACS. The dashed red diagonal line indicates the line of no discrimination (AUC = 0.50).

To evaluate the discriminatory ability of PC-FI, ACS morbidity score, and ASA classification in predicting clinically relevant postoperative complications, ROC curve analysis was performed. For this analysis, relevant major complications were defined as CCI > 30, while CCI ≤ 30 represented an uncomplicated or mildly complicated postoperative course. The ROC curves are illustrated in Figure 2, and the detailed AUC comparisons are summarized in Table 4. The standalone discriminative ability of the PC-FI was relatively modest in discriminative accuracy, yielding an area under the curve (AUC) of 0.63 (95% CI: 0.52–0.74). This performance profile was slightly lower than, but statistically non-inferior to, the standard preoperative ACS Morbidity Score (AUC 0.70, 95% CI: 0.59–0.81) and the standard subjective ASA classification (AUC 0.68, 95% CI: 0.56–0.80). DeLong’s test confirmed a total absence of statistically significant differences among the three distinct screening modalities. The overall performance data for the individual screening configurations are summarized in Table 4.

The optimal operational PC-FI cutoff calculated via the maximum Youden Index was 0.14, aligning with our predefined structural severity stratification thresholds. The specific diagnostic and predictive capability metrics of the PC-FI at this 0.14 threshold are fully expanded in Table 5, demonstrating moderate overall diagnostic parameters across the surgical track.

## 4. Discussion

In this retrospective cohort analysis, a higher preoperative frailty status quantified via the Primary Care Frailty Index (PC-FI) was significantly associated with an elevated continuous postoperative complication burden (CCI) and an extended post-surgical recovery timeframe (LOS) in older individuals undergoing elective major resection for colonic or gastric malignancies. Patients exhibiting moderate-to-severe baseline frailty profiles (PC-FI ≥ 0.14) experienced a significantly higher mean CCI score (25.96 vs. 16.40) and required an average of 2.46 additional inpatient hospitalization days compared to mildly frail counterparts.

However, it is crucial to interpret our statistical predictive metrics with appropriate caution. The standalone discriminative performance of the PC-FI achieved an AUC of only 0.63, which represents a strictly modest mathematical capability. While DeLong’s testing revealed no statistically significant differences when compared directly to established surgical estimation benchmarks such as the ACS Morbidity Score (AUC 0.70) or the subjective ASA status (AUC 0.68), our data explicitly demonstrate that none of these individual instruments possesses sufficient accuracy to function as a standalone, autonomous prognostic metric in clinical practice.

The major clinical utility of the PC-FI highlighted by this study resides not in individual perioperative risk calculation, but in its potential application as a simple, objective, and low-resource preoperative screening trigger. By leveraging routinely available electronic health data, this tool bypasses the logistical constraints of specialized physical performance testing, which is frequently unfeasible in fast-tracked oncologic pathways. Consequently, identifying an older surgical candidate with a PC-FI ≥ 0.14 should function as an immediate clinical alert to initiate comprehensive multidisciplinary assessment, targeted physical prehabilitation, nutritional optimization, and proactive discharge planning.

### 4.1. Methodological Limitations

The substantial limitations of this study must be clearly and transparently acknowledged to contextualize the generalizability of our findings:

Retrospective Design: The study relies heavily on historical electronic medical record extraction. Despite meticulous independent data validation protocols, retrospective extraction introduces an inherent risk of information bias and documentation gaps concerning soft functional or behavioral health deficits.

Small Sample Size and Power Constraints: The final cohort was limited to 92 patients, which restricts overall statistical power and increases the probability of Type II errors. Specifically, the lack of statistical significance observed in our pairwise ROC comparisons may stem directly from sample size limitations rather than genuine performance equivalence among the tools. Furthermore, the low absolute count of mortality events (*n* = 5) completely precludes robust multivariable survival modeling.

### 4.2. Selection Bias

Due to institutional pre-screening guidelines, our study population intentionally excluded robust or completely non-frail older patients (PC-FI ≤ 0.07). Truncating the lower spectrum of systemic risk automatically limits our ability to evaluate the comprehensive discriminative capability of the index across an unselected, all-inclusive older general surgical population.

Clinical Heterogeneity: The cohort combines patients treated for both colonic and gastric malignancies. These separate diseases entail fundamentally distinct baseline nutritional impacts, require highly heterogeneous surgical interventions (e.g., right colectomy vs. radical total gastrectomy), and are associated with widely divergent inherent technical risk and postoperative recovery pathways.

### 4.3. Residual Confounding

Due to the retrospective nature of the database, several critical perioperative risk parameters could not be integrated into our multivariable adjusting models. Most notably, we lacked standardized data regarding computed-tomography-derived sarcopenia metrics (e.g., psoas muscle area), granular baseline nutritional markers (e.g., prealbumin), acute preoperative inflammatory biomarkers, specific intraoperative complexity scores, or detailed fluid-management variables. The presence of residual confounding from these unmeasured variables cannot be excluded.

### 4.4. Future Perspectives

Future prospective, multicenter studies encompassing the full spectrum of baseline frailty—including robust individuals—are warranted to fully define the external validity and comprehensive discriminative accuracy of the PC-FI across unselected surgical populations. Furthermore, future research should investigate whether tracking dynamic shifts in the PC-FI during structured multimodal prehabilitation pathways can actively modulate and mitigate downstream postoperative morbidity.

## 5. Conclusions

In conclusion, our findings demonstrate that a higher preoperative frailty burden quantified via the Primary Care Frailty Index is significantly associated with an increased cumulative postoperative complication burden and prolonged hospital stay following major gastrointestinal oncologic resection. However, given its strictly modest individual discriminative accuracy (AUC 0.63), the PC-FI does not possess sufficient predictive capacity to be utilized as an independent, standalone perioperative risk prediction tool or prognostic instrument. Its true principal clinical value lies in its role as an accessible, rapid, and low-resource first-line screening surrogate to flag vulnerable older individuals and trigger early, multi-specialty perioperative optimization within a structured multidisciplinary care framework.

## Figures and Tables

**Table 1:** Baseline Characteristics of the Study Population.

Variable	Value (*n* = 92)
Age (years), Mean (Median)	79.07 (79)
Female/Male, *n* (%)	50 (54.3%)/42 (45.7%)
Colorectal/Gastric Cancer, *n* (%)	80 (87.0%)/12 (13.0%)
Laparoscopic/Open Approach, *n* (%)	68 (73.9%)/24 (26.1%)
ASA Class II/III/IV, *n* (%)	36 (39.1%)/53 (57.6%)/2 (2.2%) (ASA I: 1 patient)
Mean PC-FI (Median, IQR)	0.17 (0.16, 0.12–0.20)
Mild Frailty (PC-FI 0.07–0.13), *n* (%)	42 (45.7%)
Moderate-to-Severe Frailty (PC-FI ≥ 0.14), *n* (%)	50 (54.3%)

**Table 2:** Comparison Between Mild and Moderate-to-Severe Frailty Groups.

Feature	Mild Frailty (*n* = 42)	Moderate-to-Severe Frailty (*n* = 50)	*p*-Value
Age, median (IQR)	76.0 (72–81)	81.5 (77–85)	<0.001
Laparoscopic Approach, *n* (%)	29 (69%)	39 (78%)	0.400
ASA Class III–IV, *n* (%)	13 (31%)	42 (84%)	<0.001
Charlson Comorbidity Index, median	6.0	7.0	<0.001
ACS Morbidity Risk Estimate, median	9.4%	15.8%	<0.001
ACS Mortality Risk Estimate, median	0.4%	2.4%	<0.001
Postoperative Hospital Stay (LOS), mean	7.43 days	9.89 days	<0.05
Comprehensive Complication Index (CCI), mean	16.40	25.96	<0.05

**Table 3:** Multivariable Logistic Regression Model Predicting Major Postoperative Complications (CCI > 30).

Covariates	Adjusted OR (aOR)	95% CI	*p*-Value
PC-FI (per 0.05 increase)	1.32	1.04–1.71	0.024
Age (per year)	1.03	0.95–1.12	0.410
ASA Class (III–IV vs. II)	1.84	0.76–4.45	0.178
ACS Morbidity Risk Score	1.05	0.98–1.13	0.145

**Table 4:** ROC and AUC Analysis for Major Complications (CCI > 30).

Screening Score	AUC	95% CI	Pairwise Comparison	*p*-Value
PC-FI	0.63	0.52–0.74	PC-FI vs. ACS	0.770
ACS Morbidity Score	0.70	0.59–0.81	PC-FI vs. ASA	0.810
ASA Classification	0.68	0.56–0.80	ACS vs. ASA	0.870

**Table 5:** Predictive Operational Ability of the PC-FI at the Optimal 0.14 Cut-off.

Operational Parameter	Value	95% CI
AUC (Overall Accuracy Profile)	0.64	0.53–0.75
Sensitivity	68%	51–82%
Specificity	62%	46–76%
Positive Predictive Value	68%	51–82%
Negative Predictive Value	62%	46–76%

## Data Availability

The data presented in this study are not publicly available due to privacy and ethical restrictions related to patient clinical information. De-identified data may be available from the corresponding author upon reasonable request, subject to institutional approval and compliance with applicable data protection regulations.

## Abbreviations

The following abbreviations are used in this manuscript:

ACS	American College of Surgeons
ASA	American Society of Anesthesiologists
AUC	Area Under the Curve
BMI	Body Mass Index
CCI	Comprehensive Complication Index
IQR	Interquartile Range
LOS	Length of Stay
PC-FI	Primary Care Frailty Index
ROC	Receiver Operating Characteristic
